# A case control study of the relationship between persistent serum creatine kinase elevation and polyneuropathy

**DOI:** 10.1038/s41598-024-64555-x

**Published:** 2024-06-15

**Authors:** Svein I. Bekkelund, Karin Abeler, Hallvard Lilleng, Sissel Løseth

**Affiliations:** 1 Department of Clinical Medicine, Faculty of Health Science, UiT, The Arctic University of Norway, Tromsø, Norway; 2 Department of Neurology and Neurophysiology, University Hospital of North Norway, Tromsø, Norway

**Keywords:** Creatine kinase, HyperCKemia, Nerve conduction study, Electromyography, Polyneuropathy, Neuronal physiology, Peripheral nervous system, Kinases

## Abstract

Creatine kinase (CK) has been associated with neuropathy, but the mechanisms are uncertain. We hypothesized that peripheral nerve function is impaired in subjects with persistent CK elevation (hyperCKemia) compared to age- and sex matched controls in a general population. The participants were recruited from the population based Tromsø study in Norway. Neuropathy impairment score (NIS), nerve conduction studies (NCS) and electromyography (EMG) in subjects with persistent hyperCKemia (*n* = 113; 51 men, 62 women) and controls (*n* = 128; 61 men, 67 women) were performed. The hyperCKemia group had higher NIS score than the controls (*p* = 0.050). NCS of the tibial nerve showed decreased compound motor action potential amplitude (*p* < 0.001), decreased motor conduction velocity (*p* < 0.001) and increased F-wave latency (*p* = 0.044). Also, reduced sensory amplitudes of the median, ulnar, and sural nerves were found. EMG showed significantly increased average motor unit potential amplitude in all examined muscles. CK correlated positively with glycated hemoglobin and non-fasting glucose in the hyperCKemia group, although not when controlled for covariates. The length dependent polyneuropathy demonstrated in the hyperCKemia group is unexplained, but CK leakage and involvement of glucose metabolism are speculated on.

## Introduction

Creatine kinase (CK) is an intracellular enzyme that catalyzes energy reactions by splitting phosphate from creatine and adenosine diphosphate (ADP) to create adenosine triphosphate (ATP). CK is distributed in cells with high or fluctuating energy requirement such as skeletal, cardiac smooth muscle, brain and neuronal and kidney and a shuttle system between ATP production and ATP utilization sites plays a crucial role in the CK metabolism complexity^1^. Elevated CK that occurs in about 5% of the population, normalized in 70% of the cases after a standardized control test^2^ while the prevalence of persistent CK elevation (hyperCKemia) was 1.3% in a Caucasian population^3^. A report from the same population study demonstrated a positive association between CK and glycated hemoglobin (HbA1c) in non-diabetic subjects^4^.

In a retrospective study of 450 patients with peripheral neuropathy, 20% of the subjects had elevated CK^5^. After excluding patients with concomitant risk factors for CK elevation, isolated polyneuropathy was identified in 31 (6.9%)^5^. In another study, CK was markedly elevated in 8 patients with muscle cramps but without presence of neuropathic symptoms. Diagnostic evaluation showed that they had asymptomatic small-fiber neuropathy^6^. Elevated CK is previously reported in different samples of heterogenic neurogenic disorders. These include post-polio patients with neuromuscular deficits^7^, spinobulbar muscular atrophy (SBMA)^8^, amyotrophic lateral sclerosis (ALS)^9^ and acute inflammatory demyelinating polyneuropathy (AIDP) where elevated CK has been associated with axonal degeneration and poorer prognosis^10^. Whether CK plays an independent role in the pathogenesis of the diseases or is merely a surrogate marker without neurogenic biological effect, is an open question.

Since CK has been related to idiopathic polyneuropathy as well as certain specific neurogenic disorders in cross-sectional studies, a controlled study aiming to investigate associations between persistent hyperCKemia and peripheral nerve function in subjects randomly selected from a general population without evidence of current or previous neurological disorders is warranted and therefore conducted in a case control design.

## Materials and methods

### Study population and design

**Figure 1:**
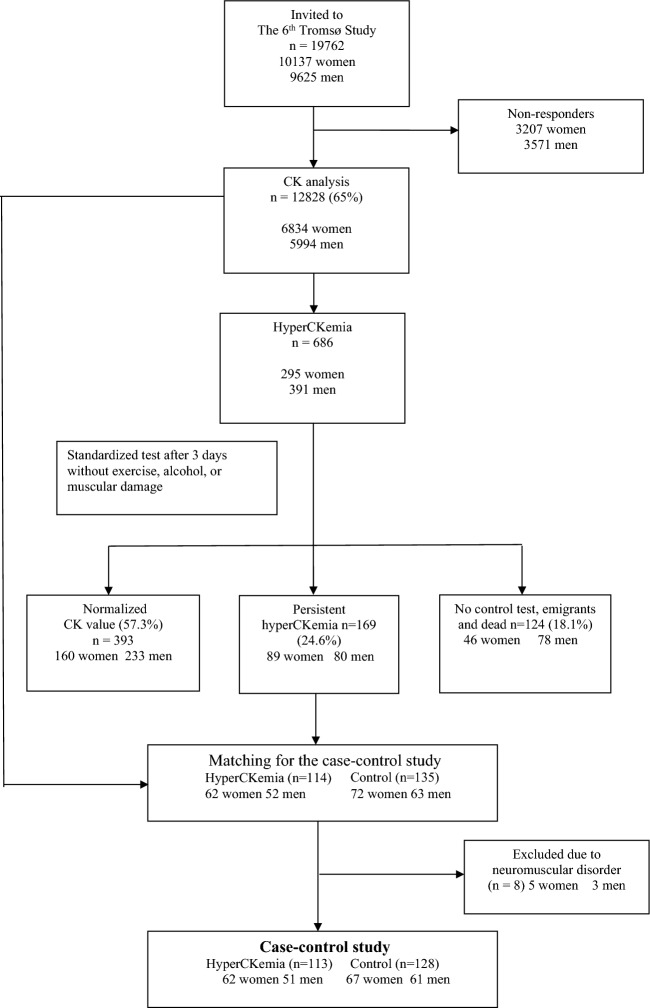
Flowchart of participant inclusion in persistent hyperCKemia and controls.

Figure 1 shows a flow chart of eligible patients, dropouts, and reasons for dropping out of the study. Selection criteria were: (1) men and women aged 30–87 years and (2) no evidence of previous or ongoing neuromuscular disorders. Participants in both groups were selected from the 6th Tromsø Study, a longitudinal population-based study that started in 1974. Novel participants and stratified groups from the 4th Tromsø study; a 10% random sample from age groups 30–39, all participants aged 40–42 and 60–87, and a 40% random sample of subjects aged 43–59 years were invited and data continuously collected from 1 October 2007 to 19 September 2008 in 12,984 participants^11^. The majority were Caucasians; 87.3% Norwegians, 1.6% Sami, 1.3% Finnish, 2.2% of other ethnicities and 7.6% without information about ethnicity^12^.

Participants in the case control study were recruited from the Tromsø study where CK was analyzed in 12,828 subjects. After performing a standardized controlled CK test and matching the groups by age and sex, 113 subjects with persistent hyperCKemia and 128 with persistent normal CK were included in the case–control study. All were Caucasians. During clinical examination, 8 participants were excluded due to previous or ongoing neuromuscular disorders: polyneuropathy (n = 4) including one with Churg-Strauss syndrome, polymyositis (n = 1), myasthenia gravis (n = 1), congenital polio (n = 1), facioscapulohumeral muscular dystrophy (n = 1) (Fig. 1). In total, one with polyneuropathy was excluded from the control group and 7 from the case group.

We used the Scandinavian NORIP references to define hyperCKemia (men < 50 years: 50–400 U/L, men ≥ 50 years: 40–280 U/L and women: 35–210 U/L)^13^. A total of 686/12,984 (5.3%) subjects with high CK were invited to a standardized control test after being instructed to refrain from muscle strain, trauma, physiotherapy, acupuncture, and alcohol intake 3 days prior to the CK control test. Persistent hyperCKemia was diagnosed in 169 subjects (Fig. 1). Age- and sex matched controls within 30–50 percentile of the CK references from the sample of participants with normal CK were selected and matched if the ages were within 5-year group intervals, and then recruited to the case control study. A study consultant (AKK) administered the participant logistics to ensure a double-blind setting throughout the study (i.e., neither the participants nor the examiners were aware of the CK levels).

### Clinical and biochemical parameters

The participants were consulted at Department of Neurology and Neurophysiology, University Hospital of North Norway. A comprehensive evaluation was performed to detect information about general health, diseases and risk factors associated with neuropathy and myopathy. This included current smoking, history of coronary heart disease and kidney disease, presence of hypertension, diabetes mellitus, hypothyroidism, use of medication such as lipid lowering drugs, and waist and hip circumference used to calculate waist-hip ratio. Furthermore, background variables associated with elevated CK were selected in accordance with literature recommendations^14–17^. Data collection included questionnaires, clinical and neurophysiological examinations, and biological sampling. Additional information and confirmation of medical history reported by the participants were obtained from our hospitals record’s system. Some of the demographic and clinical information used in the study were selected from questionnaires used in the Tromsø Study.

The following parameters were obtained from a structured interview: Ethnicity (“Caucasian”, “African”, “Other”), previous or current peripheral nerve disease and myopathy, cancer, systemic metabolic disease, haematological disorders, thyroid and parathyroid disease and liver disease, malignant hyperthermia or malignant neuroleptic syndrome, physical exercise (mild activity; activity without sweating or breathlessness < 3 h per week, high leisure physical exercise; activity with sweating or breathlessness ≥ 3 h per week, Alcohol Use Disorders Identification Test (AUDIT) where a score ≥ 8 indicates harmful alcohol consumption^18^, use of medication reported to be associated with elevated CK; lipid lowering drugs (statins)^19^, betablockers^20^, clozapine^21^ and isotretinoin^22^. Known muscle disease among first and second degree relatives, presence of muscle pain, stiffness or cramps the last two weeks and fatigue severity scale were recorded^23^.

Height and weight were measured standardized with light clothing without shoes, and body mass index (BMI) calculated as weight (kg) divided by height squared (m^2^). Blood pressure was measured in supine position by using a digital monitor (A&D Model UA-779; A&D Instruments Ltd, Abingdon, Oxon, UK). Hand grip strength (kPa) was measured in dominant hand by a Martin vigorimeter; Elmed Inc., Addison, IL, USA^24^. The best of three efforts was recorded. Knee extension of the dominant leg was tested using a Cybex NORM dynamometer (CSMI, Norwood, MA, USA). After a short, standardized warm up, the participants performed 3 subsequent knee extension and flexions. The average Nm of 3 tests were recorded. Medical Research Council (MRC)-sum score ranging from 0 to 60 assessing global muscle strength on both sides^25^ and a complete clinical neurological examination was performed using Neuropathy Impairment Score (NIS), a clinical neuropathy instrument with a total score ranging from 0 (normal) to 240. Sensation and reflexes were graded from 0 (normal) to 2 (absent) and muscle weakness from 0 (normal nerve function) to 4 (paralyses)^26^. Total NIS score and numbers/frequencies with distal sensory loss, impaired ankle reflexes and distal muscle weakness were recorded. Clinical examinations were performed by experienced neurologists (HL, SIB).

Serum CK was automatically analyzed within 6 h from sample withdrawals at the Department of Clinical Chemistry, University Hospital of North Norway (CK-NAC, Roche Diagnostics, Mannheim, Germany) with an analytic variation coefficient of ≤ 1.6%. All biochemical tests were analyzed at the same laboratory and included liver transaminases, lactate dehydrogenase, serum lipids, high-sensitive C-reactive protein (hs-CRP), serum creatinine, non-fasting serum glucose, glycated hemoglobin A1c (HbA1c-%), vitamin B12, folate and thyroid hormone levels^4,11^.

### Neurophysiological examinations

Nerve conduction studies (NCS) and electromyography (EMG) were conducted by experienced neurophysiologists (KA, SL) in the neurophysiology lab at Tromsø University Hospital using Keypoint Classic equipment (Medtronic, Skovlunde, Denmark) according to standard techniques^27^. The NCS examinations were performed unilaterally on the dominant side. Surface electrodes were utilized for motor and sensory stimulation and registration. Motor NCS included the median, ulnar, and tibial nerves, and sensory NCS the median and ulnar nerves (orthodromic stimulation) and the sural nerve (antidromic stimulation). For motor nerves the following parameters were analyzed: distal latency, amplitude, conduction velocity (CV), and minimum F-M wave latency of 20 supramaximal stimuli, and for sensory nerves: distal latencies, amplitudes, and CVs. EMG examinations with concentric needle were performed in extensor digitorum communis, vastus lateralis and anterior tibial muscles unilaterally on the dominant side. Spontaneous activity with muscle at rest was assessed (fibrillation potentials and positive sharp waves in x out of 10 needle positions, fasciculation potentials, complex repetitive discharges, and myotonic discharges). With slight voluntary muscle contraction, 20 unique motor unit potentials (MUPs) were collected, and amplitude, duration and polyphasia (%) were recorded. At last, interference pattern with gradually increasing muscle contraction were recorded (10 different measurements of turns amplitude with references from the manufacturer).

### Statistical analysis

Background variables and endpoints were evaluated by inspection of histograms which showed right-sided skewness for NIS and CK. Non-parametric test was therefore used for NIS and CK was analyzed log transformed. Descriptive data are presented as mean and standard deviation (SD), median (IQR) or number and frequency. Student´s t-test, Mann–Whitney U test respectively χ^2^-test were used to assess statistical differences between means, medians and frequencies of data and Pearson correlation coefficient used to measure strength and direction between continuous variables while Spearman rank correlation was used to measure associations between NIS score and neurophysiological parameters. Variables statistical significantly associated with persistent hyperCKemia were included in logistic regression analyses to adjust for confounders. In cases with colinearity, like ALT and AST, the one with the strongest association was selected. The analyses were performed repeatedly in model 1 (tibial nerve cMAP amplitude) and model 2 (tibial nerve MNCV). Two-sided *p* < 0.05 was considered statistically significant. All analyses were conducted by SPSS software (Statistical Package for Social Science INC, Chicago, Illinois, USA), version 29.

### Ethical declaration

This study was conducted according to the Declaration of Helsinki and was approved by theThe Regional Ethical Committee for research (approval number: REK NORD 11/2008). Informed consent was obtained from all subjects.

### Consent to participate

All subjects in the study gave their written consent prior to inclusion in the study.

## Results

### Subject characteristics

In total, 113/169 (66.9%) subjects with persistent hyperCKemia in this general population sample were included in the case control study (Fig. 1). Demographic and clinical characteristics are presented in Table 1. Mean age was 59.7 years in the hyperCKemia group and 59.4 years in the control group. None of the participants reported presence of neuromuscular disorders among first- or second-degree relatives.

**Table 1:** Demographic and clinical characteristics in subjects with and without persistent hyperCKemia. Data are presented as mean (SD) or numbers (%).

	Case Persistent hyperCKemia (n = 113)	Control Persistent normal CK (n = 128)	*p* value
Men	51 (45.1)	61 (47.7)	0.697
Age (years)	59.7 (9.3) (IQR 32–78)	59.4 (10.1) (IQR 30–77)	0.815
Height (m^2^)	169.8 (9.4)	169.6 (8.9)	0.870
Weight (kg)	81.0 (14.6)	77.8 (13.8)	0.087
BMI (kg/m^2^)	27.8 (4.0)	27.1 (3.9)	0.164
Obesity (BMI ≥ 30 kg/m^2^)	30 (26.5)	30 (23.4)	0.551
Waist-Hip ratio	0.92 (0.07)	0.92 (0.08)	0.851
High leisure physical exercise^a^	25 (22.1)	23 (18.0)	0.250
AUDIT	2.71 (2.77)	2.69 (2.11)	0.948
AUDIT ≥ 8	4 (3.5)	3 (2.3)	0.709
History of coronary heart disease	4 (3.5)	9 (7.0)	0.392
History of kidney disease	5 (4.4)	5 (3.9)	1.0
Hypertension	48 (42.5)	44 (34.4)	0.144
Use of betablocker	11 (9.7)	17 (13.3)	0.546
Diabetes mellitus	5 (4.4)	2 (1.2)	0.256
Hypothyroidism	14 (12.4)	7 (5.5)	0.066
Use of lipid lowering drugs	25 (22.1)	31 (24.2)	0.878
Duration of lipid lowering drugs use (months)	75.1 (67.9)	58.7 (52.0)	0.318
Systolic BP (mmHg)	138.8 (23.0)	139.0 (22.1)	0.442
Diastolic BP (mmHg)	79.5 (9.5)	77.9 (10.3)	0.206
S-CK (U/L) total^b^	365.5 (137.3)	91.2 (16.7)	< 0.001
S-CK (U/L) men	442.1 (148.6)	107.4 (8.2)	< 0.001
S-CK (U/L) women	305.0 (90.6)	76.7 (4.9)	< 0.001
S-ALT (U/L)	26.8 (10.1)	24.0 (9.6)	0.038
S-AST (U/L)	28.4 (5.6)	23.5 (4.9)	< 0.001
S-LDH (U/L)	183.9 (18.0)	158.5 (20.7)	< 0.001
S-Total kolesterol (mmol/L)	5.60 (1.1)	5.66 (1.1)	0.645
S-HDL (mmol/L)	1.53 (0.4)	1.50 (0.4)	0.647
S-LDL (mmol/L)	3.54 (1.0)	3.59 (0.9)	0.708
Hs-CRP (mg/dL)	2.29 (3.1)	2.48 (4.7)	0.710
S-Creatinine (µmol/L)	71.3 (15.4)	69.1 (14.0)	0.258
S-Glucose (mmol/L)	5.3 (1.0)	5.2 (0.8)	0.404
S-HbA1c (%)	5.7 (0.6)	5.6 (0.4)	0.214
S-HbA1c (%) ≥ 6.5	12 (10.6)	5 (3.9)	0.076
B12 (pmol/L)	380.2 (129.9)	381.9 (127.4)	0.919
Folat (nmol/L)	18.9 (7.1)	18.3 (6.9)	0.548
TSH (mU/L)	2.0 (0.9)	1.9 (0.8)	0.411
FT3 (pmol/L)	4.7 (0.7)	4.7 (0.6)	0.479
FT4 (pmol/L)	15.3 (2.3)	15.2 (2.1)	0.815

*ALT* alanine aminotransferase, *AST* aspartate aminotransferase, *AUDIT* Alcohol Use Disorders Identification Test, *BMI* body mass index, *BP* blood pressure, *CK* creatine kinase, *hs-CRP* high-sensitive C-reactive protein, *HbA1c* glycated haemoglobin, *IQR* inter quartile range, *LDH* lactate dehydrogenase, *TSH* thyroid stimulating hormone, ^a^Leisure physical activity with sweating or breathlessness ≥ 3 h per week; ^b^CK control test after refraining from muscle strain, trauma, physiotherapy, acupuncture, and alcohol intake 3 days prior to phlebotomy.

### Clinical and laboratory examinations

S-CK, S-ALT, S-AST and S-LDH levels were significantly elevated in the hyperCKemia group compared to the control group, but no disease markers associated with risk of polyneuropathy such as blood sugar abnormalities or B12 deficiency in any group were detected (Table 1). Number (%) of participants using statins and betablockers (drugs theoretically associated with elevated CK) were similar between the groups. Neither was S-CK levels different between subgroups using any of these drugs. Thus, mean CK in statin users (n = 56; 23.1%) was 208.6 U/L (SD 144.9, max CK 589) compared to 221.4 (SD 173.1, max CK 1046) U/L in non-statin users, *p* = 0.618. Mean CK in users of betablockers (n = 28; 11.6%) was 210.0 U/L (SD 175.8, max CK 737) and 219.3 (SD 165.2, max CK 1046) U/L in the others, *p* = 0.786. Table 2 shows more neuropathy symptoms (higher NIS score) in the hyperCKemia group while muscle symptoms, hand grip- and knee extensor power showed similar outcome between the case and control groups (Table 2).

**Table 2:** Muscular symptoms, muscular functions, and neuropathy impairment score in subjects with persistent hyperCKemia and age and sex matched controls. Data are presented as mean (SD) or median (IQR).

	Case Persistent hyperCKemia (n = 113)	Control Persistent normal CK (n = 128)	*p* value
Symptoms			
Medical Research Council sum score	59.8 (0.7)	59.0 (7.2)	0.224
Muscle cramps	22 (19.5)	28 (21.9)	0.750
Muscle pain	54 (47.8)	52 (40.6)	0.297
Muscle weakness	18 (15.9)	20 (15.6)	1.0
Muscle stiffness	49 (43.4)	41 (32.0)	0.110
Fatigue severity scale	12.6 (9.5)	14.5 (12.0)	0.181
Quantitative clinical findings			
Neuropathy impairment score	5 (IQR 0–46)	4 (IQR 0–60)	0.050
Sensory loss, distally	83 (73.5)	93 (72.7)	0.659
Ankle reflex impaired	16 (14.2)	12 (9.4)	0.232
Distal muscle weakness	3 (2.7)	4 (3.1)	0.412
Hand grip, dominant hand (kPa)	91.1 (24.9)	87.8 (22.0)	0.283
Knee extensor strength, dominant (Nm)	116.1 (45.0)	112.0 (38.6)	0.443

*CK* creatine kinase, *IQR* inter quartile range.

### Neurophysiological examinations

Tables 3 and 4 present findings from NCS and EMG examinations. NCS of the tibial nerve showed reduced cMAP amplitude, reduced MNCV and prolonged F-wave latency in the hyperCKemia group compared to controls (Table 3). Additionally, reduced SNAP amplitudes of the median, ulnar, and sural nerves were found (Table 3). EMG showed significantly increased average motor unit potential amplitude in all examined muscles in the case group compared to controls (Table 4). There were significant correlations between higher NIS score and reduced cMAP amplitude (*r* = − 0.508, *p* < 0.001) and MNCV (*r* = − 0.487, *p* < 0.001) of the tibial nerve. Furthermore, NIS correlated positively with average motor unit potential amplitudes in all the examined muscles in the hyperCKemia group (EDC, *r* = 0.389, *p* < 0.001; Deltoid, r = 0.276, *p* = 0.004; VL, r = 0.317, *p* < 0.001 and TA, 0.359, *p* < 0.001). There were positive correlations between CK and HbA1c-% (r = 0.336, *p* = 0.032) and non-fasting glucose (r = 0.531, *p* < 0.001) in the hyperCKemia group. Tibial nerve cMAP amp and MNCV, but not glucose parameters were independently related to persistent hyperCKemia in a multivariate analysis (Table 5).

**Table 3:** Nerve conduction study in subjects with persistent hyperCKemia and age and sex matched controls. Data are presented as mean (SD).

	Case Persistent hyperCKemia (n = 113)	Control Persistent normal CK (n = 128)	*p *value
Median nerve cMAP amp (mV)	7.3 (2.2)	7.0 (1.8)	0.213
Median nerve motor distal latency (ms)	3.9 (0.6)	3.6 (0.5)	< 0.001
Median nerve MNCV (m/s)	55.5 (4.7)	56.3 (4.8)	0.226
Median nerve F-wave latency (ms)	24.3 (2.4)	24.0 (2.3)	0.321
Median nerve SNAP amp (µV)*	46.0 (32.4)	52.0 (30.0)	0.137
Median nerve sensory distal latency (ms)	2.1 (0.4)	2.0 (0.3)	0.002
Median nerve SNCV (m/s)	52.4 (9.1)	55.9 (8.8)	0.003
Ulnar nerve cMAP amp (mV)	8.1 (1.8)	7.7 (1.9)	0.103
Ulnar nerve MNCV (m/s)	57.4 (7.0)	58.2 (8.4)	0.456
Ulnar nerve F-wave latency (ms)	25.1 (3.6)	24.6 (3.5)	0.329
Ulnar nerve SNAP amp (µV)	17.5 (9.7)	20.2 (11.7)	0.060
Ulnar nerve SNCV (m/s)	58.7 (9.1)	59.0 (9.9)	0.843
Tibial nerve cMAP amp (mV)	6.6 (4.3)	9.3 (4.5)	< 0.001
Tibial nerve MNCV (m/s)	41.9 (7.8)	44.8 (4.3)	< 0.001
Tibial nerve F-wave latency (ms)	51.4 (8.1)	49.6 (6.1)	0.044
Sural nerve SNAP amp (µV)*	7.8 (6.0)	9.5 (5.8)	0.023
Sural nerve SNCV (m/s)	48.0 (9.3)	49.4 (6.0)	0.173

*CK* creatine kinase, *cMAP amp* compound muscle action potential amplitude, *MNCV* motor nerve conduction velocity, *SNAP amp* sensory nerve action potential amplitude, *SNCV* sensory nerve conduction velocity, *P-values calculated from log-transformed data.

**Table 4:** Electromyographic findings in subjects with persistent hyperCKemia and age and sex matched controls. Data are presented as mean (SD) or number (%).

	Case Persistent hyperCKemia (n = 113)	Control Persistent normal CK (n = 128)	*p *value
EDC amplitude of MUP (mV)	832.2 (341.7)	745.9 (273.7)	0.031
EDC duration of MUP (ms)	10.1 (1.6)	9.9 (1.4)	0.261
EDC polyphasia (%)	16.5 (12.4)	18.6 (13.1)	0.202
Deltoid amplitude of MUP (mV)	706.9 (475.3)	602.7 (171.0)	0.022
Deltoid duration of MUP (ms)	10.7 (2.2)	12.1 (1.6)	0.373
Deltoid polyphasia (%)	10.3 (8.7)	12.9 (9.4)	0.033
VL amplitude of MUP (mV)	1224.5 (656.8)	1045.7 (489.7)	0.017
VL duration of MUP (ms)	12.7 (2.20)	12.1 (1.6)	0.029
VL polyphasia (%)	12.2 (9.8)	12.0 (10.1)	0.873
TA amplitude of MUP (mV)	1494.7 (1079.1)	1119.1 (593.5)	< 0.001
TA duration of MUP (ms)	12.0 (2.7)	11.2 (1.5)	0.005
TA polyphasia (%)	19.5 (11.8)	21.1 (11.6)	0.305
Spontaneous activity	7 (6.2)	2 (1.6)	NA

*CK* creatine kinase, *EDC* extensor digitorum communis, *MUP* motor unit potential, *NA* not applicable, *TA* tibialis anterior, *VL* vastus lateralis.

**Table 5:** Logistic regression models for tibial nerve amplitude (model 1) and conduction velocity (model 2) with variables associated with persistent hyperCKemia.

	Model 1		Model 2	
	ß (95% CI)	*P* value	ß (95% CI)	*p* value
Tibial nerve cMAP amp (mV)	1.11 (1.01–1.29)	0.024		
Tibial nerve MNCV (m/s)			0.90 (0.83–0.99)	0.021
Age (years)	0.94 (0.90–0.99)	0.008	0.93 (0.88–0.97)	< 0.001
Sex	1.04 (0.44–2.44)	0.932	0.89 (0.37–2.18)	0.804
S-HbA1c (%)	1.68 (0.74–3.81)	0.216	1.31 (0.54–3.19)	0.546
Neuropathy impairment score	1.02 (0.98–1.07)	0.324	1.01 (0.97–1.06)	0.535
S-AST (U/L)	1.21 (1.07–1.36)	0.003	1.20 (1.07–1.36)	0.004
S-LDH (U/L)	1.07 (1.00–1.10)	< 0.001	1.07 (1.05–1.10)	< 0.001

*AST* aspartate aminotransferase, *CI* confidence interval, *cMAP amp* compound muscle action potential amplitude, *HbA1c* glycated haemoglobin, *LDH* lactate dehydrogenase, *MNCV* motor nerve conduction velocity.

## Discussion

In two otherwise comparable groups with and without persistent hyperCKemia, neuropathic impairment score and impaired neurophysiological responses in peripheral nerves suggesting polyneuropathy were demonstrated in the hyperCKemia group. The findings were most consistent for motor nerves, and particularly the tibial nerve. A positive relationship between CK and blood sugar (HbA1c and non-fasting glucose) in the hyperCKemia group indicate glucose metabolism to be involved in the process.

An association between hyperCKemia and neuropathic changes demonstrated by NCS in the present study support the view of a biological relationship between elevated CK and peripheral nerve dysfunction. In an American retrospective study in patients with peripheral neuropathy from 2021, a 3-fold higher occurrence of muscle cramps was reported in patients with concomitant hyperCKemia^5^. In comparison, 20% of the subjects in the present study reported muscle cramps regardless of CK group. Thus, muscle cramps have by others been associated with both small fiber and large fiber neuropathy^6,28,29^. A positive relationship between neuropathic symptom score and neurophysiological parameters strengthens the view of an ongoing disease process. Several methodological issues make comparisons difficult, especially regarding the case control design, recruitment of presumptive healthy subjects from a general population and use of quantitative neurophysiological examinations for outcome measures. Moreover, performing a standardized controlled CK-analysis after incidental detection of elevated CK is important to reduce confounding effects of muscular activity^3^.

In a retrospective cohort of 100 subjects with incidentally detected hyperCKemia (mean CK: 1410 U/L), 13 had neurophysiological confirmed neurogenic changes including 8 with concomitant myogenic abnormalities^30^. Neither that study nor others have identified any CK-related neurogenic mechanism explaining such findings, however. Neither have recent studies reporting elevated CK in subgroups of AIDP patients with axonal degeneration documented any pathophysiological mechanism^10,31^. The present study outcomes with reduced amplitude and conduction velocity along with prolonged F-wave latency found in the tibial nerve can neither exclude axonal nor demyelinating processes, but such a division of the NCS findings is uncertain in a non-diseased population, and particularly changes in the amplitude sizes should be interpreted cautiously^32^. A few more participants in the hyperCKemia group had spontaneous activity on EMG in the present study, but too few to allow for valid statistical analyses. Another finding was a significant increase in amplitudes of MUAPs in all studied muscles in the hyperCKemia group while duration of MUAP and polyphasic potentials showed similar results by QEMG examinations (Table 4). Although a moderate increase in MUAP amplitudes may be seen as an unspecific sign rather than part of a neurodegenerative process, it may also be associated with muscle cell hypertrophy hypothetically reflecting an effect of CK^33,34^.

CK correlated positively with HbA1c and glucose in the hyperCKemia group, although the statistically significant association disappeared in a multivariate analysis. This association confirms previous population-based data in non-diabetic subjects^4^, but contrasts an Asian study that showed a negative association between CK and HbA1c in a general population^35^. Elevated CK may occur in diabetes^36^ and a possible biological link is previous reported relationships between CK elevation, insulin resistance and obesity, all being associated with muscle fiber type 2b activity^37^. There is a known relationship between polyneuropathy and HbA1c^38^, and impaired NCS responses may be present before neuropathic symptoms appear in diabetic subjects^39,40^. Also, polyneuropathy is reported in prediabetes (HbA1c 5.7–6.4)^41^, but not at lower levels of HbA1c like the data from the present study. Hypothetically, leakage of CK due to increased plasmalemma permeability caused by neuropathy-related denervation of muscle cells is a mechanism to consider. Previous electron microscopy studies have shown increased permeability of denervated muscle cell membranes^42,43^. Furthermore, elevated serum CK has been found in rats with denervated skeletal muscle cells^44^.

Follow-up data in CK related neuropathy is largely lacking. Thus, one subject with polyneuropathy was detected during 7.2 years mean follow-up time after initial diagnostic work-up of 31 subjects with idiopathic hyperCKemia^45^ but no case of polyneuropathy was found in a long-term follow-up study (7.5 years) among 55/93 initially unclassified subjects with hyperCKemia^46^. Limitations to the present study include the retrospective design, lack of common peroneal and superficial peroneal recordings and lack of data on small nerve fiber function. Performing two CK tests including one standardized test do not prove presence of persistent hyperCKemia. On the other hand, comparing the data with an age- and sex matched control group in a predominantly Caucasian general population are main advantages since important CK-confounders are thereby controlled for.

## Conclusion

In this case control study, subclinical neuropathic findings by neurophysiological examinations were found in otherwise healthy individuals with persistent hyperCKemia in a general population indicating an independent relationship between elevated CK and polyneuropathy. The finding is largely unexplained although glucose parameters correlated positively with CK. Further CK vs. neuropathy studies should compare neurophysiological parameters with higher CK-levels and include examinations of peroneal nerves and small nerve fibers. Possible etiological factors such as plasmalemma CK leakage and abnormal glucose metabolism should be further investigated.

## Data Availability

Due to ethical and legal restrictions, data is only available upon request to the Tromsø Study. Any enquiries should be sent to the Institutional Data Access committee of The Tromsø study, Department of Community Medicine, Faculty of Health Sciences, UiT- The Arctic University of Tromsø (tromsous@uit.no).
